# Long-term follow-up of memory impairment after third ventricle colloid cyst surgery

**DOI:** 10.1016/j.bas.2025.104301

**Published:** 2025-06-18

**Authors:** Matteus Zywczyk, Per Hellström, Dan Farahmand, Sanna Pääaho, Magnus Tisell

**Affiliations:** Department of Neurosurgery, Institute of Neuroscience and Physiology, Sahlgrenska Academy, University of Gothenburg, Gothenburg, Sweden

**Keywords:** Colloid cyst, Memory impairment, Neurosurgery, Psychometrics, Long term effect

## Abstract

**Introduction:**

Colloid cysts are rare benign but potentially lethal intracranial tumors. Treatment is surgical with open microsurgery (OS) or endoscopic surgery (ES). Postoperative memory disturbance due to fornix injury is a dreaded complication. Memory problems may severely affect life quality.

**Research question:**

To investigate the long-term memory function after colloid cyst-surgery.

**Materials and methods:**

Twenty-two patients (mean age 45 years) previously operated with OS (n = 15) or ES (n = 7) underwent objective memory testing, clinical interviews including self-estimated Quality of Life (QoL) and subjective memory questionnaires after a mean follow-up time of 7.6 years.

**Results:**

Subnormal objective memory performance was on average seen in 38 % of the patients. Cyst diameter correlated negatively to objective mean memory score (multiple R = 0.57, p = 0.005). All patients with a cyst diameter exceeding 15 mm (n = 5) had memory performance below the 10th percentile. Memory scores were similar in endoscopically and microsurgically treated patients.

Objective memory performance was not correlated to subjective memory performance, sex, preoperative hydrocephalus or acute presentation.

QoL ratings and depression scores were strongly correlated with the assessments of subjective memory impairment, but not with objective memory performance.

There was no recurrence cyst on MRI.

**Discussion and conclusion:**

Large preoperative cysts had worse memory outcomes. Self-reported QoL and depression did not correlate with objective memory performance but with subjective memory problems. Anxiety was not associated with objective or subjective memory performance. No difference was found in objective memory performance between patients who underwent OS or ES.

## Highlights

• Large preoperative cysts were linked to worse long-term memory outcomes.
•Objective memory performance was not correlated with subjective memory performance.
•Subjective memory was correlated with self-rated quality of life and depression.
•Memory performance was similar after open microsurgery and endoscopic surgery.

## 1 Introduction

Colloid cysts are rare, benign intracranial cystic lesions typically located in the anterior roof of the third ventricle of the brain (Blumenfeld, 2010), accounting for between 0.3 and 2 % of all radiological findings of brain tumors (Macdonald et al., 2005; Ahmed et al., 2018; Jeffree and Besser, 2001). There is no definitive consensus on the etiology, but various theories exist, one of which proposes that the cysts originate from the paraphysis, a third ventricle structure derived from the neuroectoderm that is transiently present in human embryos (Macdonald et al., 2005; Nagaraju et al., 2010).

The cysts are benign in nature and sometimes discovered incidentally. However, should a colloid cyst reach sufficient size, it may obstruct cerebrospinal fluid circulation partially or completely, leading to obstructive hydrocephalus presenting with progressive or intermittent headaches of varying intensity as the main symptom (Yadav et al., 2015; Spears, 2004; Young and Silberstein, 1997; Sabanci et al., 2017). Other symptoms may be nausea, vomiting, cognitive changes, visual blurring secondary to papilledema, rapid loss of consciousness and, in severe cases, even death (Yadav et al., 2015). Colloid cysts can be present at any age, but the majority of reported patients are between 30 and 60 years of age (Yadav et al., 2015).

In case of surgical intervention, two management strategies currently exist: shunt surgery or excision of the cyst. Because shunt surgery is associated with a high rate of long-term complications, and excision provides possibility of definitive treatment, the latter is usually preferred. There are two major widely used excision techniques - open microsurgery with craniotomy (OS), and the less invasive endoscopic method (ES)(Yadav et al., 2015).

Indications for surgery are usually based on cyst size, (usually >7–10 mm in diameter, depending on local guidelines), and symptomatology. Cysts are known to occasionally recur (Sheikh et al., 2014), and as of yet there are no guidelines regarding long-term follow-up.

One large meta-analysis, including a total of 1278 patients, by Sheikh et al., concluded that OS is associated with a higher rate of complete resection, a lower rate of recurrence, and fewer reoperations than ES, but that the rate of morbidity is higher. (Sheikh et al., 2014). Other studies and reviews have reached similar results and conclusions, and also indicate that ES generally leads to shorter post-operative stay lengths (Sabanci et al., 2017; Horn et al., 2007; Grondin et al., 2007; Farahmand et al., 2023).

One of the more debilitating complications that might result from colloid cyst surgery is transient or permanent memory impairment (Nitta and Symon, 1985; Soleman and Guzman, 2020). The exact mechanism is unclear, but it may be a consequence of the location of colloid cysts in proximity to the fornices of the limbic system, structures known to be important for learning and memory formation. The fornices are crucial in processing long term memory connecting the hippocampus and the mammillary bodies. Colloid cysts of the third ventricle are typically situated in the roof of the third ventricle in adjunction to the two crura of the fornix. These columns might be variously displaced by the colloid cyst depending on growth pattern. Two studies, with a total of 18 cases, that examined the relation between MRI-determined fornix damage and memory loss as measured by psychometric tests, both concluded that bilateral interruption of the fornix resulted in anterograde amnesia in all cases (Aggleton et al., 2000; McMackin et al., 1995). The main type of memory affected seems to be recall memory (Fuentes et al., 2022). Impaired memory functioning is associated with a negative effect on the quality of life (QoL)(Salans et al., 2021).

Roth et al. tested memory function in 23 colloid cyst patients pre-operatively, three months after surgery, and one year after surgery, concluding that memory function among other cognitive functions improved and remained stable after surgery (Roth et al., 2019). Still, long-term data on both objectively assessed memory function and QoL after surgery on colloid cysts remain scarce. This study aimed to investigate long-term results of memory function as well as QoL in patients with colloid cysts after surgical cyst extirpation.

## 2 Patients and methods

**Fig. 1:**
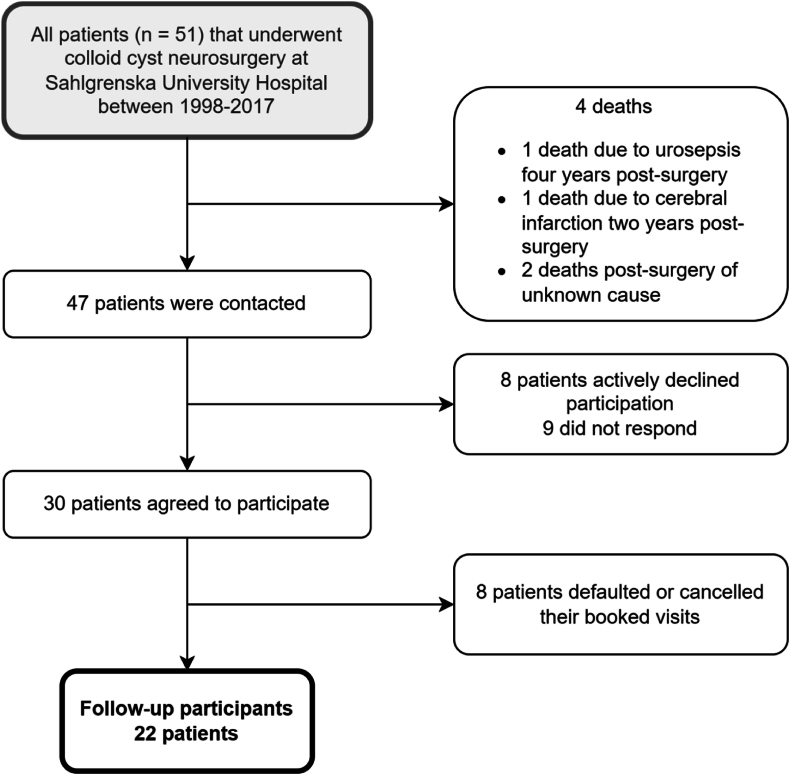
Flowchart of patients and dropouts.

Fifty-one consecutive patients with colloid cysts from Western Sweden (1.8 million inhabitants) were surgically treated for colloid cysts between 1998 and 2016. All patients were contacted and 22 agreed to participate in a long term follow up (Fig. 1). Hospital records were reviewed to gather pre- and postoperative data (Table 1). All patients operated with open microsurgery were operated with interhemispherically approach during this time period. All neuro-endoscopic procedures were performed from the non-dominant hemisphere taking right- or left handedness into account.

**Table 1:** Epidemiological data of tested patients from journals. OS = open interhemispherically microsurgery, ES = endoscopic surgery, VP = ventriculoperitoneal.

Patient No.	Sex	Age at surgery	Years between surgery and follow-up	Cyst size (mm)	Clinically recorded symptoms	Co-morbidity	Surgical technique	Post-operative complications
**1**	M	58	2	15	Vertigo, radiological growth	none	ES	none
**2**	M	42	3	9	Headache	none	ES	none
**3**	F	42	3	5	Headache, vertigo	none	ES	none
**4**	M	44	3	9	Headache, vertigo	none	ES	none
**5**	M	39	7	9	Headache, vertigo, nausea, hydrocephalus	none	ES	none
**6**	M	55	8	17	Headache, severe memory impairment, hydrocephalus	none	ES	none
**7**	M	58	9	20	Gait disturbance, memory impairment, urinary incontinence, hydrocephalus	COPD	ES	CSF leakage, infection, postoperative VP-shunt
**8**	F	26	4	6	Headache, radiological growth	none	OS	none
**9**	F	34	5	7	Headache, memory impairment, dysphasia	none	OS	none
**10**	F	26	5	15	Headache, fatigue, blurred vision, hydrocephalus	none	OS	Atrial fibrillation
**11**	M	30	6	18	Headache, seizure	none	OS	none
**12**	M	41	7	9	Headache, dysphasia, hydrocephalus	Depression, anxiety, migraine	OS	none
**13**	F	29	7	6	Headache	none	OS	Hallucinosis
**14**	F	20	9	17	Headache, nausea, balance disturbance, syncope, papilledema, hydrocephalus	none	OS	none
**15**	M	42	11	13	Headache, hydrocephalus	Hypertension	OS	none
**16**	M	32	11	10	Headache, tinnitus, facial paresthesia, hydrocephalus	Cyst excision 1993	OS	none
**17**	M	26	12	9	Headache, nausea, impaired consciousness, hydrocephalus	none	OS	none
**18**	F	46	12	15	Headache, memory impairment, impaired consciousness, hydrocephalus	none	OS	Stroke secondary to brain herniation
**19**	F	23	13	15	Headache, nausea, impaired consciousness, hydrocephalus	none	OS	none
**20**	M	43	15	20	Headache, nausea, memory impairment, balance disturbance, impaired consciousness	Previous VP-shunt due to cyst	OS	none
**21**	M	37	17	10	Headache, hydrocephalus	none	OS	none
**22**	M	17	20	7	Headache, severe memory impairment, hydrocephalus	none	OS	none

The clinical long-term follow-up consisted of three parts; firstly, a standardized clinical interview according to a case report form (covering preoperative comorbidity, symptoms before surgery, perioperative complications and remaining postoperative symptoms, as well as occupational status), followed by memory evaluation and finally life quality questionnaires. All the components of the interviews were administered by the first author (MZ) according to a verbal and written set of instructions by an experienced neuropsychologist (PH).

There had been no systematic objective memory assessment pre-operatively or post-operatively of the patients prior to this follow up. The memory assessment was divided into two parts; one for subjective (self-reported) memory performance and one for objective (psychometrically tested) memory performance. Subjective memory performance was assessed using a questionnaire, the EMQ-R, that consists of 13 items examining subjective memory impairment related to daily activities and living (Royle and Lincoln, 2008). The score ranges from 0 (no subjective memory problems) to 52 (very severe impairment). The questionnaire was translated from English to Swedish by author PH. Afterwards, the patients were tested with four objective memory tests. The Rey Auditory Verbal Learning Test™ (RAVLT), evaluating verbal learning and retention capacity (Schmidt, 1996), the Brief Visuospatial Memory Test–Revised™ (BVMT-R) measuring visuospatial memory function (Benedict, 1997),the Word Pair Test quantifying verbal paired retention and, finally, the Image Pairs Test examining visual paired learning and recollection. The two latter test were developed in-house (appendix 1).

The EQ-5D-5L (Herdman et al., 2011) was used for measuring QoL. EQ-5D consists of 5 items in 5 dimensions of life quality as well as a visual-analogue scale for self-rating of the subjective level of health. For assessing depression and anxiety the Hospital Anxiety and Depression Scale (HADS), with seven items for depression and seven items for anxiety, was used. (Zigmond and Snaith, 1983). For HADS, a cut-off of 8 points both for depression and for anxiety has been used previously (Bjelland et al., 2002).

Additionally, all patients were offered and accepted an MRI of the brain to detect any recurrent colloid cyst.

Prior to subject recruitment, the study was approved by the regional ethics board of Gothenburg (diary no. 309-18), and all subjects were informed of the study by letter and telephone. Those who participated gave informed, written consent after reading a study information sheet. The study was performed in alignment with the principles of the Declaration of Helsinki.

## 3 Statistics

Data was collected in printed forms and questionnaires and exported to SPSS version 24 for subsequent analysis. The Mann-Whitney *U* test was used for group comparisons. To make a composite score of all the six objective memory tests, the raw values were transformed to standardized T-scores (i.e., with a mean of 50 and a standard deviation of 10), whereafter a mean T-score was calculated. Linear regression analysis was used to calculate correlations between T-scores and clinical parameters.

## 4 Results

The participants were examined at a mean time of 9 years after surgery (range 2.3–20.3 years). The mean age at surgery was 37 years, 64 % (n = 14) were male, 68 % (n = 15) were treated with open microsurgery, and the mean cyst diameter was 12 mm. The three most common symptoms were headache (91 %), preoperative memory impairment (27 %) and nausea (23 %). Fifty-nine percent had hydrocephalus as indication for surgery.

The EMQ-R test showed a wide variety of subjective memory problems with a mean impairment score of 15. of maximum 52 (range 1–52, SD = 13).

**Fig. 2:**
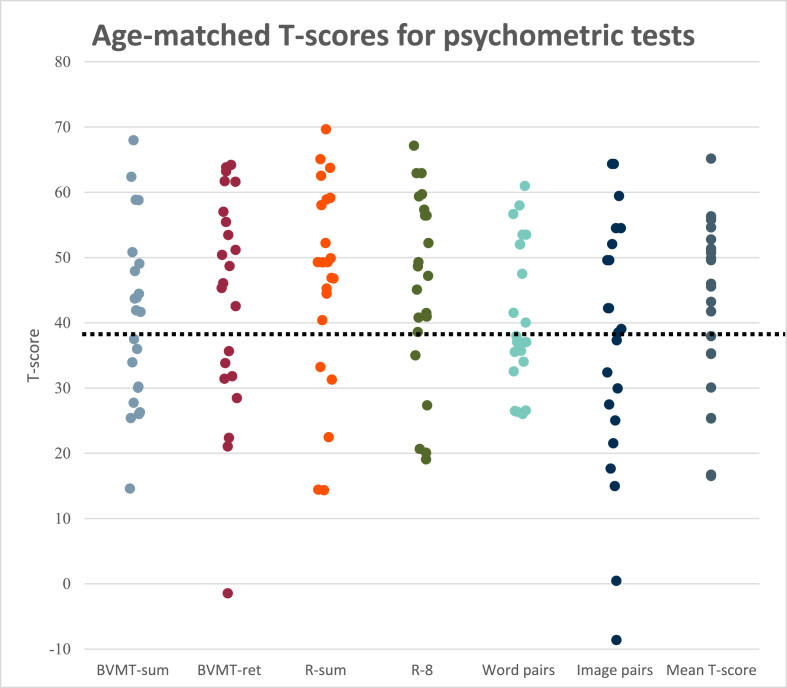
Clustered boxplots showing the patient's results in the form of T-scores for the objective memory tests. T-scores are standardized to a standard population of the same age with T-scores <38 (below the dotted line) corresponding to the 10th percentile of the general population.

The results of the four memory tests are shown in Fig. 2. The patients had subnormal performance (<10th percentile) as compared to age-matched reference values, with impairments seen in 23–55 % (mean: 38 %) of the patients depending on what test was used. The worst performance was seen in the paired learning tests, with 55 % of patients showing below par performance for both tests. We did not find any significant difference in objective memory function between the patients who underwent OS and those subjected to ES in any of the administered memory tests or regarding the mean T-scores (*p* > 0.05 for all variables, Mann-Whitney *U* test).

**Fig. 3:**
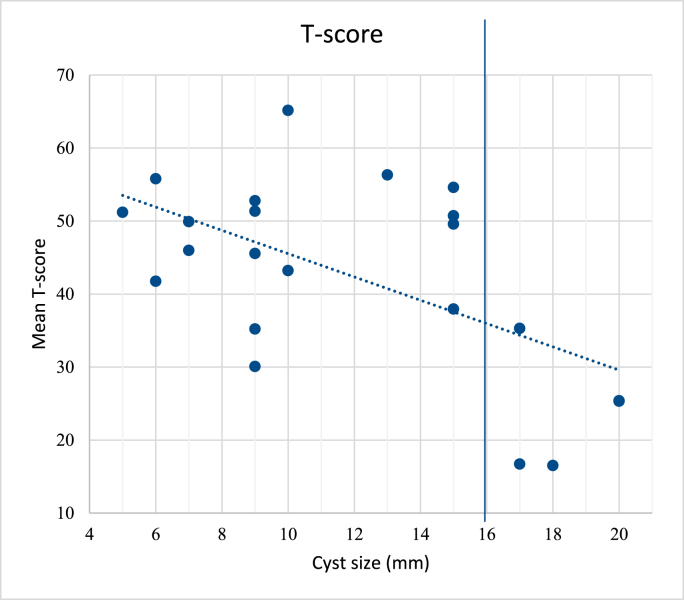
Plot between cyst size in millimetres and the mean T-score (p = 0.005, linear regression analysis).

A significant negative correlation was found between cyst diameter and mean objective memory score (multiple R = 0.57, p = 0.005)(Fig. 3). All patients with cysts larger than 15 mm (n = 5) had an objective memory performance below the 10th percentile cut-off level. There was no significant correlation between either subjective memory (as measured by EMQ-R-sum) or age and mean T scores (p > 0.05, linear regression analysis). Further, neither sex, preoperative hydrocephalus, nor acute presentation were associated with mean T scores (p > 0.05). At trend level (p = 0.055), patients with preoperatively diagnosed memory impairment (according to hospital records) had worse long-term memory T-scores.

QoL as reported in EQ5-5D was strongly correlated with the subjective memory impairment score of EMQ-R (p < 0.01, multiple R = 0.73) but not with the objective memory mean T-score (p = 0.57).

Likewise, the HADS depression score was strongly correlated with the subjective memory score (p < 0.01), but not with the objective memory performance mean T-score (p = 0.45). The HADS anxiety score, finally, was not correlated with either subjective (p = 0.12) or objective memory (p = 0.23).

None of the participants (n = 22) had a recurrent cyst on MRI at the long-term follow up.

## 5 Discussion

We found that the patients with the largest colloid cysts and preoperatively clinically recorded memory impairment had the highest risk of post-operative long-term memory deficits regardless of operation technique. This is in accordance with previous knowledge that progressively overstretched or compressed neural tissue is more prone to irreversible damage and worse post-operative neurological outcome (Baro et al., 2021; Kreβner et al., 2018).

As shown by regression analysis in this study, a larger preoperative cyst diameter was linked to worse memory outcome as previously reported by Dhandapani et al. (2021) This may be due to a higher compressive stress on adjacent brain tissues, including the fornices, or due to increased difficulty with larger cysts during surgery. Depending on the technique and angle of approach used, different areas of the brain can be injured during surgery to create a trajectory to the colloid cysts in the third ventricle, but the most sensitive part of the surgery might be the dissection of the cyst from the surrounding tissues including the fornices. While ES might be less traumatic to the cranium, dura mater and cortex on the way down to the colloid cyst, it shares the same difficulties as OS concerning the detachment of the colloid cyst from adjacent structures. Thus, the risk of fornix damage might be the same regardless of surgical method and more related to the size of the cyst, an assumption that is at least functionally supported by the lack of differences in objective memory scores between OS and ES patients.

However, as we have no objective preoperative memory testing. we can not differ between irreversible preoperative damage caused by a large cyst and surgically caused memory deficits.

In our study, the subjective memory ratings were not significantly correlated with objective memory performance, a result aligned with previous research showing weak if any correlations between subjective and objective cognitive (including memory) performance (Crumley et al., 2014; Burmester et al., 2016). Correspondingly, we did not find that objective memory performance affected either QoL or the anxiety and depression scores. Conversely, however, subjectively reported memory impairment was related both to QoL and depression. Among our patients, then, it appears that self-rated memory problems reflected general or psychological well-being rather than actual capacity to learn and recall information.

As third ventricle colloid cysts are of rare occurrence, we recommend future multicenter, prospective register-based studies. Besides radiological data, surgical information and complication registration, such studies should include pre- and postoperative tests of memory functions as well as QoL assessments. In addition one should include more detailed imaging radiological of the relevant anatomical structures related to the cysts.

### 5.1 Strengths and limitations

A major strength of this study was the long-term follow-up including objective psychometric memory testing in combination with subjective measures of memory impairment and quality of life assessment. To date, data on long-term outcomes are scarce. The number of patients in the present study is relatively large considering the rarity of the disease (Sheikh et al., 2014). They were also included consecutively and from a defined geographical area and thus representative of the local population of Western Sweden.

The follow-up was standardized and extensive. To reduce the risk of being led astray by a single test outlier in memory testing, the objective assessment of memory was evaluated using a mean T-score, comprising the results of six different scores.

A limitation was that pre-operative information on memory impairment was retrospectively gathered from clinical data and did not include objective tests, and since patients may have reduced awareness when self-evaluating memory function, this may be underreported at acute presentation. Ideally, patients should be tested before and after surgery in future studies, although – due to the nature of the disease – this may be impossible in emergency cases.

Another limitation is the rather large drop-out rate. Patients who want to participate might be both better and worse than the average.

## 6 Conclusions

In this study, large preoperative cysts were significantly linked to worse long-term memory outcomes. There was no difference in objective memory performance between patients subjected to the two different surgical methods.

Self-reported quality of life and depression were not correlated with objective memory performance but were associated with subjective memory problems. Anxiety had no correlation with either objective or subjective memory performance.

We suggest that pre- and postoperative memory testing is included perioperatively in colloid cyst surgery for accurate assessment of surgical outcomes.

## Declaration of competing interest

The authors declare that they have no known competing financial interests or personal relationships that could have appeared to influence the work reported in this paper.

## Appendix 1 Instructions for the Word Pairs Test and the Image Pairs Test (Translated from Swedish)

### Instructions for the Word Pairs Test (Power Point administration)

PRESS refers to pressing the spacebar, pg dn, down arrow or right arrow, or whatever else scrolls forward in Power Point.

Start with a blank screen.

“This test is called Word Pairs (PRESS).
You will see a series of different pairs consisting of two words on the screen. The first word will be written in white text (PRESS) and the second will be green (PRESS).
Your task is to remember the second word, the one that is green.
They are both related in such a way that they belong to the same overall category.
(PRESS) Now you will see two practice examples. Try to remember which green word is related to the white word in each pair.
(PRESS) Skin
(PRESS) Pupil. The common category here is body parts, but as you can see, we have chosen two words that are not obviously connected.
(PRESS) Palm tree
(PRESS) Carrot. Here the common category is plants, but as before, these are two words that have no obvious connection.
(PRESS) Ok, which word went with skin?
(PRESS) And which word went with palm tree?”

Test 1.

“Now you will see 10 consecutive word pairs. When you have seen all word pairs, you will see the white words again, but in a different order, and then I want you to try to remember what the green word was in each pair.” (PRESS)

When the picture with the question mark appears, say:

“Now you will see the white words again, one at a time, and for each word, you should try to remember the green word that went with the white one. Remember that the words belong to the same overall category even if the connection may seem far-fetched.”

Test 2.

Now you will see 10 new word pairs. You should do exactly the same as before. So try to remember the green word in each pair.

?

When the picture with the question mark appears, say:

"Now you will see the white words again, one at a time, and for each word you should try to remember the green word that went with the white one. Remember that the words belong to the same overall category even if the connection may seem far-fetched."

Test 3.

Now you will see the last 10 word pairs. Do exactly as before. Try to remember the green word in each pair.

?

When the picture with the question mark appears, say:

“Now you will see the white words again, one at a time, and for each word you should try to remember the green word that went with the white one. Remember that the words belong to the same overall category even if the connection may seem far-fetched.”

Instructions for the Image Pairs Test (Power Point administration)

PRESS refers to pressing the spacebar, pg dn, down arrow or right arrow, or whatever else scrolls forward in PowerPoint.

Start with a blank screen.

“This test is called Image Pairs (PRESS).

You will see a series of different image pairs on the screen. Each pair consists of two identical images. One of the images will be placed in the center of the screen, like this (PRESS).

The second image, the twin image, will be placed in one of the following eight locations on the edge of the screen:

In one of the corners (PRESS 4 TIMES),

directly to the left (PRESS)

or directly to the right (PRESS) of the image in the center,

directly above (PRESS)

or directly below (PRESS) the image in the center (PRESS).

It is only in one of these eight places (count the positions and PRESS 8 times) on the edge of the screen that the twin image will be placed, never between them like this (PRESS)

and never closer to the image in the center like that (PRESS).

Your task is to remember where the twin image was placed.

Now you will do two practice examples. Try to remember where the twin images are placed.

(PRESS) (the Mallard duck)

(PRESS) (the church)

(PRESS) Ok, where was the other Mallard duck placed?

(PRESS) And where was the other church placed?

Test 1.

“Now you will see 15 consecutive image pairs. After you have seen all image pairs, you will see the center images again, but in a different order, and then I want you to try to recall where the twin images were placed.”

Test 2 “Now you will see another 15 image pairs. Try to remember where the twin images are placed, just like before.”
